# Issues in pediatric vaccine-preventable diseases in low- to middle-income countries

**DOI:** 10.1080/21645515.2016.1181243

**Published:** 2016-06-20

**Authors:** Ghassan Dbaibo, Vladimir Tatochenko, Peter Wutzler

**Affiliations:** a Center for Infectious Diseases Research, Department of Pediatrics and Adolescent Medicine, American University of Beirut Medical Center, Riad El Solh, Beirut, Lebanon; b Child Health Research Centre, Moscow, Russia; c Friedrich Schiller University of Jena, Institute of Virology and Antiviral Therapy, Jena, Germany

**Keywords:** barriers, developing countries, Pediatric vaccines, vaccine-preventable disease, vaccine implementation

## Abstract

The highest burden of pediatric vaccine-preventable disease is found in developing nations where resource constraints pose the greatest challenge, impacting disease diagnosis and surveillance as well as the implementation of large scale vaccination programmes. In November 2012, a Working Group Meeting convened in Casablanca to describe and discuss the status with respect to 8 vaccine-preventable diseases (pertussis, pneumococcal disease, measles-mumps-rubella-varicella (MMRV), rotavirus and meningococcal meningitis) to identify and consider ways of overcoming obstacles to pediatric vaccine implementation. Experts from Europe, Russia, the Commonwealth of Independent States, the Middle East, Africa and South East Asia participated in the meeting. A range of region-specific needs and barriers to uptake were discussed. The aim of this article is to provide a summary of the ongoing status with respect to pediatric vaccine preventable disease in the countries represented, and the experts' opinions and recommendations with respect to pediatric vaccine implementation.

## Introduction

The meeting, sponsored by GlaxoSmithKline Biologicals S.A., was held to debate key issues in vaccine-preventable diseases in low to middle income countries and was attended by 7 faculty members and 29 experts representing Germany, the Netherlands, Russia, Commonwealth of Independent States (CIS; Kazakhstan, Ukraine), Middle East (Bahrain, Lebanon, Saudi Arabia), Africa (Ivory Coast, Egypt, Tunisia, Kenya) and South East Asia (India, Pakistan, Bangladesh). The meeting involved presentations, discussion sessions and interactive workshops across 8 different pediatric vaccine-preventable disease areas. The key topics of interest were the changing epidemiology of pertussis and optimum vaccination schedules, the impact of pneumococcal conjugated vaccines, MMRV vaccination and safety, rotavirus vaccine effectiveness and safety and the implementation of meningococcal conjugated vaccines.

Key objectives of this meeting were to describe the vaccination status of 8 vaccine-preventable diseases in the regions represented, to identify and consider ways of overcoming obstacles to pediatric vaccine implementation and provide information to allow policymakers to make informed decisions about pediatric vaccine implementation. This article provides a summary of these discussion topics in addition to the experts' opinions and recommendations.

## Shifting pertussis epidemiology and the need for new vaccine strategies

Pertussis is one of the leading causes of vaccine-preventable deaths worldwide.^1^ Despite the dramatic impact of vaccination,^2^ mortality due to pertussis remains significant and most deaths are in infants who are too young to be protected by vaccination. During the 1990s, 66% of pertussis deaths reported to the Centers for Disease Control and Prevention (CDC) were in infants 0–1 month of age.^3^ Establishing the burden of pertussis disease is challenging due to a low awareness of pertussis among physicians, the absence of effective surveillance systems and the variability of diagnostic methods used. The increase in the reported incidence of pertussis cases in the US and Australia since 2007 highlights that in the post-vaccine era the disease is shifting from younger to older age groups.

Because of this changing epidemiology, there is a need for new strategies to improve vaccination against pertussis with the aim of increasing protection for the most vulnerable, i.e. newborns, and lowering the overall health burden of pertussis in the population.^4^ The current recommendations for booster vaccination in Europe and the US are summarised in Table 1.^4-6^

**Table 1.** Summary of current booster recommendations for pertussis-containing vaccines in Europe^4^ and the US.^5,6^

	ACIP recommendations (US)				
COPE recommendations (Europe)	Cocooning	Children	Adolescents	Adults	Pregnant women
Adolescents (10–17 y) should receive a single dose of combined reduced-antigen-content tetanus-diphtheria-acellular pertussis (dTap) vaccine instead of dT, irrespective of a complete primary vaccination schedule #The cocooning strategy (vaccinating close contacts of newborns with dTap) should continue until immunization coverage in adults is sufficient for herd protection	Adolescents and adults who have or anticipate having close contact with an infant aged <12 m should receive a single dose of Tdap to protect against pertussis if they have not received Tdap previously	Children aged 7–10 y who are not fully vaccinated against pertussis* and for whom no contraindication to pertussis vaccine exists should receive a single dose of Tdap. Those never vaccinated against tetanus, diphtheria, or pertussis or who have unknown vaccination status should receive a series of 3 vaccinations containing tetanus and diphtheria toxoids. The first of these 3 doses should be Tdap	Adolescents aged 11–18 y who have completed the recommended childhood DTP/DTaP vaccination series and adults aged 19 through 64 y should receive a single dose of Tdap. Adolescents should preferably receive Tdap at the 11 to 12 y old preventive health-care visit.	Adults aged 19 through 64 y who have completed the recommended childhood DTP/DTaP vaccination series should receive a single dose of TdapAdults aged 65 y and older who have or anticipate having close contact with an infant aged less than 12 m should receive a single dose of Tdap. #Other adults ages 65 y and older may be given a single dose of Tdap.	During pregnancy, optimal timing for Tdap administration is between 27 and 36 wks gestation, although Tdap may be given at any time during pregnancy. For women not previously vaccinated with Tdap, if Tdap is not administered during pregnancy, it should be administered immediately postpartum.

COPE, Consensus on Pertussis Booster Vaccination in Europe; ACIP, Advisory Committee on Immunization Practices. General guidance relating to booster vaccination only is shown. For the full recommendations refer to Zepp 2011, CDC 2011 and CDC 2013b.

While both European and US advisory bodies recommend vaccination of adolescents, differences are seen in the recommendations for adults and pregnant women.^4-6^ In February 2013, the Advisory Committee on Immunization Practices (ACIP) updated their recommendations to include reduced antigen concentration tetanus, diphtheria, acellular pertussis (Tdap) vaccination during every pregnancy; and to recommend the use of cocooning.^6^

### Diagnosis

In Russia diagnosis is primarily clinical and laboratory confirmation takes place by bacteriology, serology and PCR. In Kazakhstan/Ukraine (CIS), diagnosis involves use of a standard clinical case definition and PCR while in the MENA region methods range from a clinical diagnosis through to a full definitive laboratory definition, although the case definition used is variable. In the Ivory Coast/Tunisia, diagnosis is primarily clinical, with very limited use of laboratory diagnosis and in South East Asia it is primarily clinical and uses the WHO definition together with lymphocytosis.

### Surveillance

In Russia, where surveillance is carried out, the incidence of pertussis was 3.4 cases/100,000 in 2010, 3.3 cases/100,000 in 2011 and 5.05 cases/100,000 in 2012,^7^ but when modern diagnostic methods are used, morbidity figures increase 4–5-fold. In some countries in the MENA region notification of pertussis cases is mandatory and active surveillance is performed.

In the Ivory Coast/Tunisia there is mandatory reporting of disease only in children <5 years, but the disease is considered to be under-reported. While surveillance is performed in Ivory Coast/Tunisia, it is based on vaccination status rather than diagnosis. Reporting of pertussis cases in South East Asia is not mandatory. Passive surveillance is performed, although a surveillance system has been established in the private sector in India.^8^

Barriers which prevent more effective diagnosis and surveillance in each region and ways to overcome them are presented in Table 2.

**Table 2.** Barriers and enablers of effective pertussis diagnosis and surveillance.

Region	Barriers	Enablers
Russia/CIS	• Lab diagnosis limited by technical complexity of bacteriological tests and high cost of PCR • Administrative barriers • Atypical cases not being diagnosed reducing accuracy of surveillance	• Use a standard case definition • Educate immunizing physicians • Increase implementation of PCR • Make use of IT for surveillance
MENA	• Lack of awareness of doctors leading to an underestimation of cases • Lack of a standard case definition • Lack of facilities for lab diagnosis • Not recognized as a public health problem due to a lack of local epidemiological studies	• Establishing a national case definition • Set up a regional surveillance network • Include vaccine preventable diseases in national reports • Identify and engage stakeholders
Africa	• Inadequate budget • Very limited facilities for laboratory diagnosis • A clinical case definition which is not pathogen specific • The possibility of atypical presentation • Inadequate reporting and feedback from the Ministry of Health	• Reduce the costs of diagnosis • Provide clinical guidance for diagnosis • Establish a reference laboratory • Improve the Ministry of Health Bulletin reporting and feedback process
South East Asia	• Difficulty of culturing the pathogen • Lack of lab infrastructure • Lack of awareness and political will • Costs of surveillance • Other healthcare priorities	• Establish national and regional reference laboratories • Conduct active surveillance • Make pertussis notifiable and enforce notification • Increase awareness and training

### Regional status of pertussis vaccination

Experts reported minimal uptake of booster vaccination in their countries to date. Barriers to vaccination include access and cost issues as the Tdap vaccine is not yet registered in some countries and vaccine funding bodies do not provide financial support for booster vaccination. In India, for example, infants receive primary vaccination and boosters are recommended in the 2nd year of life and in pre-school children^9^ but uptake of the booster doses at 2 and 5 y was reported to be negligible. Experts reported quite low (∼60–65%) and heterogeneous national vaccine coverage. In India and in most countries represented, the focus remains on implementing primary vaccination of infants and achieving high coverage.

### Implementation of additional immunization strategies and potential challenges

Given parents' concerns about the safety of whole cell pertussis (wP) vaccines, a switch to use of aP vaccines was considered to be necessary by experts in order to maintain high coverage. In Russia, only one booster vaccination is administered at 18 months (wP) and it is thought that the considerable morbidity seen in those above 6–7 y is a direct result of this.

The advantages of implementing booster doses are wider coverage ultimately leading to herd immunity and a reduction in transmission to susceptible individuals and ability to administer vaccines during school and university. However, a lack of local epidemiological data, lack of awareness of the need for boosters in some regions, poor access to the public health system or lack of contact after the second year of life in some regions make the implementation potentially challenging.

The logistical implications of vaccinating on such a wide scale must also be considered, include cold chain capacity, the cost of sustaining immunization, the availability of vaccines and the difficulty of importing large quantities of vaccines.

Other strategies for improving pertussis vaccination discussed during the meeting include extending Universal Mass Vaccination (UMV) to newborns (<2 months of age), and providing booster vaccines for pre-school children (4–6 y of age) and adults (> 18 y of age) including maternal vaccination.

However the lack of vaccines licensed for use in newborns and insufficient data on effectiveness and safety in this population are significant barriers to the potential extension of UMV to newborns. For pre-school children, key considerations include the inadequate availability of combination vaccines and the need to evaluate the potential reactogenicity of 4 or more doses of acellular pertussis (aP) vaccine. UMV in pre-school children and adolescents might also result in a shift in the disease curve to older ages and if implemented in each group alone would not generate herd immunity. In adults, key considerations are that a high level of coverage (>85%) would be needed in order to achieve herd immunity.^10^ and data are currently lacking on the duration of protection from aP vaccines and on cost-benefit.

Maternal vaccination aims to increase the immune defenses of the newborn and this could be achieved through vaccination of women of child-bearing age or during pregnancy. Key advantages of this approach are that it targets high risk groups and that the target population for vaccination is easy to access and well-motivated. Recent studies of the safety of the Tdap vaccination in pregnant women did not identify any concerning patterns of adverse events.^11-13^ Munoz *et al.* also showed that maternal immunization with Tdap resulted in high concentrations of pertussis antibodies in infants during the first 2 months of life and did not substantially alter infants immune responses to diphtheria, tetanus, acellar pertussis (DTaP) vaccine.^11^ Further large scale studies on maternal immunization with Tdap are still warranted.

Cocooning is a safe approach to protecting the most vulnerable i.e., newborns and provides extra benefits to adults including maternal immunity for the next pregnancy. It is expected to reduce transmission and decrease resurgence of cases, is cost-effective and may be an effective option in countries without universal booster vaccination strategies. However there are limited data on the contribution of disease transmission from close contacts to infants and data are lacking on cocooning effectiveness.^14,15^ Logistical challenges include having sufficient cold chain capacity and the difficulty of organizing such a strategy. Cost, poor compliance and inability to achieve herd immunity are likely to be a barrier, as are the ethical implications of selecting people for vaccination and the acceptability of this type of approach.

### Enablers for implementing additional immunization strategies

Improved surveillance is needed to provide data on pertussis incidence in order to increase disease awareness among Health Care Providers (HCP) and convince policymakers of the need for booster vaccination. Capacity building is required to improve diagnostic facilities. Some experts thought that allowing for flexibility in booster vaccine administration between 9 months and 5 y would be beneficial, others suggested promoting the use of the cocooning strategies or improving the available vaccines.

## Pneumococcal conjugate vaccine impact

An estimated 14.5 million episodes of serious pneumococcal disease occur each year in children under 5 y of age, resulting in ∼500,000 deaths, almost all of which occur in low- and middle-income countries.^16,17^

### Global status and impact of vaccination with pneumococcal conjugate vaccines

WHO recommendations for use of pneumococcal conjugate vaccines (PCVs)^18^ in childhood immunization programmes worldwide and GAVI Alliance funding have resulted in an increase in PCV introductions into national immunization programmes (NIPs), especially in lower-income countries.^19^ Countries with high childhood mortality, (mortality rate > 50 deaths per 1000 live births among children aged < 5 years) should make the introduction of multicomponent PCVs a high priority.^17^ As of November, 2014, 58% (112/194) of WHO member states had introduced PCVs.^20^

PCVs were shown to be tolerated and effective for reducing illness and deaths caused by *Streptococcus pneumoniae*.^17^ Introduction of universal mass vaccination with PCV-7 in the US, England and Wales has led to dramatic decreases in the rate of invasive pneumococcal disease (IPD), for example a 64–77% reduction in cases was seen in the US between 1999 and 2010.^21,22,23^ New generation PCVs, the 10-valent Pneumococcal non-typable *Haemophilus influenzae* protein D Conjugate Vaccine (PHiD-CV; GSK Vaccines) and the 13-valent Pneumococcal-diphtheria CRM_197_ protein Conjugate Vaccine (PCV-13; Pfizer) have been licensed based on immunogenicity data. Rates of IPD have continued to decrease since the introduction of PCV-13 in England and Wales in 2010.^23^ The Clinical Otitis Media and Pneumonia Study (COMPAS) involving PHiD-CV has provided the first efficacy data against likely bacterial community-acquired pneumonia (B-CAP) and WHO-defined consolidated CAP for a new generation pediatric PCV.^24^ The Finnish Invasive Pneumococcal Disease Vaccine Trial (FinIP) is the first randomized controlled European clinical trial of a PCV to demonstrate efficacy against IPD.^25^

PCV effectiveness is now also documented against IPD^26^ and pneumonia in a developing world setting. In January 2011, Kenya introduced PHiD-CV into the routine childhood immunization schedule. The Pneumococcal Conjugate Vaccine Impact Study (PCVIS) is a large-scale before-after study of vaccine effectiveness in residents of Kilifi County, Kenya.^27,28^ The study evaluated the total impact of PCV use in children on IPD of both vaccine and non-vaccine types, and aimed to distinguish direct from indirect effects by monitoring all individuals in a defined population for both immunization and morbidity events.

Since the introduction of routine immunization of children < 12 months in Kenya and after the first catch-up campaign there has been a marked decrease in the frequency of cases among children under 5^27^ and evidence of indirect benefits of disease reduction in older unvaccinated populations.

Impact data from the PCVIS study were considered to show great promise, though it may take several years to be confident that changes in disease frequency observed are attributable to vaccine. The absence of a significant change in the frequency of non-vaccine type cases due to serotypes replacement suggests that use of the vaccine has not led to an increase in disease related to non-vaccine serotypes. Despite these encouraging results a number of challenges remain to be overcome including maintaining uptake, ensuring vaccine financing and consolidating surveillance of disease and side effects.

### Value of efficacy and effectiveness data

The efficacy of PCVs are not in doubt and the new data on vaccine impact are exciting, including those data showing the impact on IPD during the FinIP trial, the reduction in antimicrobial prescriptions demonstrated by both PHiD-CV and PCV-7, and other indirect effects. Data on the vaccine effectiveness of both 3+1(3-primary vaccination series followed by a booster dose at different time intervals) and 2+1 ( 2-primary vaccination series followed by a booster dose) schedules were considered to be most important when describing the expected impact of pneumococcal vaccines to decision makers. The Kenyan data on the impact of PHiD-CV on weekly IPD admissions was considered to have the most relevance to vaccine policy makers in the South Asia region because many countries in both regions have a low income and a high pneumonia burden.

Efficacy against serotype-specific invasive pneumococcal disease (IPD) was considered important but requires local data on serotype prevalence to be interpreted, which is not available in the majority of countries. Serotype coverage data are available in some countries. In India, experts estimated that serotype coverage of PHiD-CV is 70% and PCV-13 is 72%. In Bangladesh, estimated coverage is 43% for PHiD-CV and 50% for PCV-13.^29^ In Russia, estimated coverage for PHiD-CV is 41% to 78.5%.^30^ In the Lebanon, estimated coverage in individuals <2, 2–5, and >60 y of age, is 53%, 74% and 45%, respectively for PHiD-CV; and 63%, 80%, and 68%, respectively for PCV-13.^31^

### Regional status of pneumococcal disease and vaccination with PCV

Experts discussed a number of factors as having an important effect on local disease rates including high population density, proximity access to health care facilities, indoor air pollution, malnutrition, host factors and antimicrobial resistance. Seasonal factors such as the Hajj pilgrimage affect disease rates in the Middle East and North Africa (MENA) region and HIV infection plays an important role in Africa.

With the exception of Kenya, PCV was not available in the NIPs of any of the regions represented at the time of the meeting. Cost of the vaccine remains a major obstacle to implementation, particularly in middle-income countries not eligible for GAVI support. Many middle income governments with limited budgets struggle to place PCV on their NIP without solid political support to help them secure the additional funds needed. In this regard, donors, such as the GAVI Alliance, may need to devise new approaches to help some of these middle-income countries to provide improved vaccine access for, at least, the lower socioeconomic segment of the population.

In the MENA region PCVs were available in the private sector at a high price and coverage was reported to be low. In India and Bangladesh, both PHiD-CV (GSK Vaccines) and PCV-13 (Pfizer) were available in the private sector at the time of the meeting and PHiD-CV has since been introduced into the NIPs of both countries in 2015; PHiD-CV became available in Pakistan in September 2012, PCV-13 is expected to be available from 2016. The introduction of PCVs into NIPs was considered important due to high under 5 morbidity and mortality rates in the regions represented, to which pneumonia is the major contributor (under 5 mortality rate: India 69, Bangladesh 53, Pakistan 87 per 1000 live births). High pneumococcal vaccine coverage was considered to be the most effective means of reducing regional disease rates with vaccine coverage of > 70% considered necessary to effectively protect the most vulnerable. In Russia, PCV UMV was introduced in 2014 where both PHiD-CV and PCV-13 are available.

### Impact of herd immunity and serotype replacement on vaccine effectiveness

Due to low or no PCV vaccine coverage in most regions represented, the importance of herd immunity and serotype replacement was generally understood, but not considered to be relevant at the time of the workshop. Appropriate vaccine schedules and availability of booster doses would be important in generating herd immunity, which is necessary to reduce transmission and protect unvaccinated individuals of other age groups. Vaccine types used and local serotype prevalence were considered important factors for serotype replacement. Longitudinal surveillance will be important following introduction of PCV into NIPs, not just to determine the direct impact but to evaluate the potential indirect effects among unvaccinated persons and vaccine-induced changes in the IPD serotype distribution. Experts considered it possible that serotype replacement could occur within 3–5 y of PCV introduction into NIPs.

## MMRV vaccination and safety

Varicella is highly infectious resulting in a disease that is usually mild to moderate in severity. In temperate climates most people are infected with varicella zoster virus, the causal agent of both varicella (chickenpox) and herpes zoster (shingles), before they reach adolescence.^32^ Varicella can result in serious complications including death; in developed countries, the overall case fatality rate from varicella is about 2–4 per 100,000 cases although the risk of death is up to 29 times higher in adults than in children.^32^ Another serious complication of varicella infection is congenital varicella syndrome which occurs in 0.4–2.0% of children born to mothers with primary varicella-zoster virus infection during the first 20 weeks of gestation.

Varicella vaccine is highly effective in preventing varicella or reducing the severity of the disease. This vaccine is available either as a monovalent formulation (V), or in combination with measles, mumps and rubella (MMRV) vaccine.

Vaccination has had a dramatic impact on the global incidence of measles, mumps and rubella (MMR) over the last 30–40 y following the introduction of the trivalent MMR vaccines and more recent drives, such as the Global Vaccine Action Plan lead by the WHO, which aims to eliminate measles and rubella in 5 WHO regions by 2020.^33-35^ However, measles and rubella remain common in many developing countries of Asia and Africa where vaccination programmes are not well established.^34,35^ The addition of a varicella component to the trivalent vaccine has the potential to have an even greater impact on the global burden of disease with the practical advantages associated with immunization with one tetravalent versus several single valency vaccines.

### Breakthrough varicella and introduction of 2-dose vaccination schedules

UMV programmes which previously included a single dose of varicella vaccine have been highly effective, nevertheless, breakthrough varicella (defined as varicella occurring > 42 d after vaccination)^36^ with a one-dose schedule is very common.^37^ Two doses lead to higher geometric mean titres of varicella antibodies, significantly higher efficacy (98.3% vs 94.4% for any varicella disease over a 10-year period, p < 0.001) and fewer breakthrough cases than one dose.^38^ The increased protection against breakthrough varicella offered by 2 doses of varicella-containing vaccine was further confirmed in a large, Phase III study of tetravalent MMRV vaccine (GSK Vaccines) administered in healthy children aged 12–18 months.^39,40^

The recommendations of ACIP of the CDC in the US with regard to the varicella vaccination schedule were revised in 2005/2006 and by the German Standing Committee on Vaccination (STIKO) in Germany, in 2010.^41,42^ Studies in the US and Germany have since confirmed the increased effectiveness of 2 doses vs. a single dose of varicella vaccine.^43-45^

### Regional status of MMR and varicella vaccination

The availability of measles, mumps, rubella and varicella-containing vaccines in the public and private sectors of the regions represented is described in Table 3. Notably, MMRV vaccine was not in use in the public sector in any of the regions represented at the time of the expert meeting. The picture across the regions is diverse; with countries using either single valency measles and/or rubella vaccines, MM+R+V vaccines, MMR vaccine alone or MMR+V vaccines. Varicella vaccination was available in the NIPs of only 2 regions. In Russia, however, regional immunization against varicella is increasing. With 1 million cases of varicella in Russia recorded in 2009, varicella is now recognized as one of the most frequent infections in children.

**Table 3.** Regional status of MMR and varicella vaccination (November 2012).

Region	Country	Public sector	Private sector
Russia		MMR: available as MM + R on a 12 m, 6 y schedule	Varicella vaccines are available in regional programmes and in the private sector
		Varicella: regional immunization is increasing	
CIS	*Ukraine*	MMR: available since 2007 using 12 m, 6 y schedule Catch-up up to 18 years since 2008 Varicella: vaccine available to high-risk groups and military recruits	Varicella vaccine available in private market
	*Kazakhstan*	MMR: since 2007 using 12 m, 6 y schedule	
		Varicella: not available	
MENA		MMR: All GCC countries have MMR at 12 m and pre-school. MMRV is not available in any country Varicella: Most GCC countries have a 2-dose schedule In Bahrain the vaccine is available in high schools and to HCPs	Varicella: Most countries have a 2-dose schedule except Oman
South East Asia	*India/Pakistan*	MMR: Measles vaccine at 9, 15 months	MMR+V at 15 m, 4–6 y (India)
			MMR+V at 13–15 m, MMRV at 4–6 y (Pakistan)
	*Bangladesh*	MMR: Measles and rubella vaccines at 9, 15 months	MMR
			Varicella single dose at 1–12 y
Africa	*Tunisia*	MMR: Measles at 15 months, 6 y, 12 y	MMR and varicella available
		Rubella exclusively for girls at 12 years	Varicella uptake very low
	*Ivory Coast*	MMR: Measles at 9 months	MMR available at 15 m, 4.5 y
			Rubella at 12 y
			Varicella uptake very low

CIS, Commonwealth of Independent States; GCC, Gulf Cooperation Council; MENA. Middle East and North Africa; MMR, measles-mumps-rubella; V, varicella

**Table 4.** Recommendations for the use of varicella-containing quadrivalent vaccines.

American Academy of Pediatrics (AAP) 2011^49^	STIKO 2012^50^
• AAP recommends that the timing of vaccine doses remains the same (12–15 m for Dose 1 and 4 to 6 yrs for Dose 2) • The first dose administered at 12–47 m can include either MMR and varicella vaccines administered separately or MMRV vaccine • Because the risk of febrile convulsions is not increased in older children who receive the second dose of MMRV, the use of MMRV is generally preferred for the first dose of vaccine administered at 48 months and older, and for Dose 2 at any age (15 m to 12 years) • It is recommended that children with a personal or family history of febrile convulsions generally should be vaccinated with separate MMR and varicella vaccines, because the risks of using MMRV in this group generally outweigh the benefits	• STIKO recommend that first dose is administered at 11–14 months and the second at 15–23 m • Separate first-dose vaccinations with MMR and monovalent varicella vaccine are recommended, due to a slightly increased risk of febrile convulsions after first-dose application of the combined MMRV vaccine

For the full recommendations refer to American Academy of Pediatrics 2011^49^ and STIKO 2012^50^

### Safety profile of quadrivalent MMRV vaccines and the assessment of benefit versus risk

ACIP was initially alerted to preliminary evidence of an increased risk of febrile convulsions after the administration of the MMRV vaccine when compared with separate MMR and varicella vaccines.^46^ Risk of febrile convulsion during Days 7–10 was found to be higher after MMRV than after MMR plus varicella vaccination with an excess risk for febrile convulsions of 4.3 per 10,000 doses (95% confidence interval: 2.6–5.6). An increased risk of febrile convulsions following MMRV vaccine administration was also reported with a relative risk of 2.20 (1.04–4.65), 5–12 d after immunization.^47^

An approximately 3–4-fold increased risk of febrile convulsion 5–12 d after the first dose of MMRV vaccine compared with MMR or MMR+V vaccination was also observed in a retrospective matched cohort study.^48^

The overall consensus of the expert panel with respect to the benefit/risk profile of MMRV vaccination was that the benefits greatly outweigh the risks and that the use of MMRV as the first dose in young children should be advocated.

### Expert review of existing recommendations for varicella-containing vaccines

The current recommendations of STIKO and the American Academy of Pediatrics (AAP) Committee on Infectious diseases for the use of varicella-containing quadrivalent vaccines are summarised in Table 4.^49,50^

They were considered to be overly cautious by some, who suggested that the actual risk is much less than the recommendations indicate and that vaccines such as whole cell DTP carried a greater risk of adverse events. Experts noted that a consequence of the introduction of these recommendations in Germany was a reduction in first dose vaccinations with varicella vaccine in the year after recommendation of 4% and 12% in the 2 surveillance regions. First-dose vaccinations for MMR (MMR or MMRV vaccine), however did not change significantly in the 2 regions.^51^ This indicates that the recommendations were not well understood and overall had a detrimental effect on vaccine uptake.

Experts believed that recommendations were lacking important data related to the use of MMRV vs. MMR + V, including morbidity and mortality, and cost-effectiveness data, and data on the optimal period between the first and second dose. Experts also felt that recommendations could be made clearer by avoiding the use of ambiguous wording. Others commented that the recommendations did not address country-specific issues (such as lack of observed febrile convulsions in India, for example) suggesting a need for country-specific recommendations. Of the available recommendations, the recommendations of STIKO were thought to most closely reflect the actual benefit/risk by some such that they may be more appropriate for use at a local level.

### Communication of the benefits versus risks of MMRV vaccination

The benefit/risk profile of MMRV vaccination was reviewed by the expert panel and suggestions made about communicating benefit/risk to parents. The opinion of the expert panel was that parents are choosing not to vaccinate because of the possibility of adverse events. It was considered likely that parents are not fully aware of the risk of disease outbreaks in the absence of vaccination against measles, mumps, rubella and varicella.

Some experts felt that the increased risk of febrile convulsions between 5–12 d after the first dose should be explained verbally to parents and that sharing responsibility with parents was important. Others noted that the risk of each adverse event was not usually explained verbally to parents for other vaccines and this might cause unnecessary stress and alarm.

## Rotavirus vaccine effectiveness and safety

The global disease burden from rotavirus was highlighted noting that rotaviruses are the leading cause of severe, dehydrating diarrhea in children aged < 5 y worldwide.^52^ Rotavirus is estimated to be responsible for ∼500,000 child deaths worldwide which equates to ∼5% of all child deaths.^52,53^ The vast majority of rotavirus-associated deaths occur in low income countries in Africa and Asia and are related to poor health care.^54^

### Rotavirus vaccines efficacy and effectiveness

Two vaccines monovalent RV1 (GSK Vaccines) and pentavalent RV5 (Merck and Co, Inc.) were available and licensed in most countries. Extensive clinical data on RV1 has been generated from the largest vaccine clinical trial program conducted by GSK, enrolling >90,000 participants from >20 countries in Latin America, Europe,^55^ Asia^56^ and Africa.^57^ In European studies, significant reductions have been reported in rotavirus gastroenteritis (RVGE)-related hospitalizations (Austria) and rotavirus-related morbidity (Germany). In the RotaBel effectiveness trial conducted in Belgium, RV1 efficacy against RVGE hospitalization was found to be 90%.^58^ Furthermore, RV1 effectiveness has also been evaluated in Latin America (Brazil, Mexico, El Salvador, Nicaragua and Panama) where it was shown to be highly effective against RVGE and RVGE hospitalization.^59,60^

In Phase III clinical trials conducted primarily in the US and Finland, efficacy of the RV5 vaccine (Merck and Co, Inc.) was 98% against severe RVGE and 94.5% in preventing hospitalization/emergency department visits related to serotypes G1-G4.^61-63^ Similar reductions were seen in studies conducted in the US, Europe, Latin America and the Caribbean.^64^ Vaccine efficacy has also been demonstrated in clinical trials conducted in Africa and Asia.^65,66^ The effectiveness of RV5 has been demonstrated in post-licensure studies conducted in Latin America, the US, Europe and Australia.^67-72^

### Rotavirus vaccine safety

RV1 has been extensively studied in clinical trials and has been shown to have a clinically acceptable safety profile.^73^

The first rotavirus vaccine was licensed by the US Food and Drug Administration (FDA) In 1998 (RRV-TV), and was withdrawn in 1999 due to an epidemiological link with intussusception (IS) with an estimated incidence of 1 per 2500–9500 vaccinees.^74-77^ A number of post-marketing studies have also been performed to evaluate the risk of IS following vaccination with RV1. In the first of these studies, there was an increased risk of IS in the first week post-Dose 1 of RV1 in Mexico (Mexico OR 5.8 [95% CI: 2.6–13.0]) and in Brazil (Brazil OR 1.4 [95% CI: 0.4–4.8]).^78^ A second study, conducted in every major hospital in Mexico (N = 221) ^79^ found a temporal association between RV1 and IS within 31 d post-Dose 1 (IS relative incidence 1.75 [95% CI: 1.24–2.48; p = 0.001]). A clustering of IS cases within 7 d of Dose 1 (IS relative incidence 6.49 [95% CI: 4.17–10.09; p < 0.01]) but not Dose 2 was also noted. This data corresponds to a risk of 3–4 additional IS cases/100,000 infants vaccinated. Another study^80^ found that for a hypothetical situation of a 9.5 million birth cohort in 14 Latin-American countries, rotavirus vaccination would prevent 144,746 hospitalizations and 4124 deaths in the first year of life and could potentially cause 172 excess hospitalizations and 10 deaths due to IS. Another observational study conducted after vaccination with RV5 (Merck and Co., Inc.) and RV1 in the US^81^ identified an increased risk of IS in the 21 day time period after the first dose of RV5, that translates into 1 to 1.5 additional cases of IS per 100,000 first doses of RV5. The US FDA approved revisions to the Prescribing Information and Patient Information for RV5 as a result of these data. Data on the risk of IS following the use of RV1 were inconclusive and did not result in changes to the Prescribing Information or Patient Information, however, revisions were made in September 2012 based on the results of the previously described study in Mexico.^79,81^

### Regional status of rotavirus vaccination and challenges to implementation

Despite the huge global disease burden from rotavirus, prevention of this disease is not seen as a priority by many of the countries represented at this meeting. This is partly due to the lack of surveillance systems in affected countries that report the true burden of rotavirus and allow decision makers to properly appreciate its importance. A number of key challenges to vaccine implementation faced by the regions are that rotavirus is low on the countries' lists of priorities compared with other diseases, the cost of the vaccine, the lack of epidemiological, surveillance and cost-effectiveness data.

At the time of the expert meeting, rotavirus vaccination was only available in NIPs in the MENA region (including Bahrain, Iraq, Qatar, Sudan and Yemen, the latter 2 through GAVI). Kenya has since commenced a UMV program.^82^ Rotavirus vaccination was more widely available in the private markets of the represented regions.

The experts concluded that the benefits of rotavirus vaccination far outweigh the potential temporal increase in IS risk. Furthermore as a risk minimization measure, the current RV1 prescribing information and patient leaflets alert HCPs and parents to monitor vaccinated infants for signs and symptoms of IS.

### The importance of benefit/risk analyses for future vaccine recommendations

The benefit/risk analyses were seen as very important for future recommendations on rotavirus vaccination. The introduction of IS surveillance, however, was noted as key to fully documenting risk in each region. Examples of risk/benefit analyses from similar countries following rotavirus vaccine introduction (such as Bangladesh) will be of considerable importance to India and Pakistan.

### Communicating the importance of rotavirus vaccination

Experts noted that hurdles to vaccination could be overcome by increasing awareness of rotavirus infections and education for physicians and the public, improving and extending rotavirus data collection, performing and sharing the results of health economic, surveillance and impact studies. Policymakers should be provided with data on disease burden, vaccine effectiveness and cost effectiveness. For the media, there is a need for real-life stories explaining the advantages of vaccination and for parents, experts noted that using educational posters and flyers in healthcare centers would be beneficial to raise awareness.

## Meningococcal conjugate vaccines and implementation

Meningococcal disease is a serious global concern, often associated with high morbidity and mortality. WHO estimates that 500,000 cases of meningococcal disease are reported each year, with case fatality rates often exceeding 10%.^83^ The burden of disease is particularly severe in the Middle East and Africa, especially in the so-called ‘meningitis belt’ in sub-Saharan Africa.^84^ The Hajj pilgrimage in the Middle East also constitutes a key factor influencing the epidemiology of meningococcal disease. For survivors of meningococcal disease, there are high rates of associated sequelae often with significant morbidity.^83^ During pandemic disease outbreaks in the meningitis belt in sub-Saharan Africa, attack rates exceed 100–800 cases per 100,000 population per year, with the highest attack rates reaching as high as 1 in 100.^83^

### Meningococcal serotype distribution

There are at least 13 known serogroups of *N. meningitidis* although most meningococcal disease worldwide is caused by just 6 of these: A, B, C, W-135, X and Y.^85^ Serogroup distribution varies considerably by age and geographical location (Table 5) although outbreaks have the potential to spread rapidly across the world.^85^

**Table 5.** Meningococcal serotype distribution and epidemiology.^85,96-98^

Serogroup	Key geographical and epidemiological characteristics
A	• Responsible for the largest and most devastating meningococcal outbreak in sub-Saharan Africa in 1996–1997 • Now rare in the US and Europe
B	• Associated with a lower incidence of disease compared to serogroup A or C • Prolonged outbreaks of disease cause significant morbidity and mortality • The most important cause of endemic disease in developed countries, causing:o 20–40% of disease in the USo up to 80% in Europe
C	• Responsible for part of the reported endemic disease and localized epidemic outbreaks in developed countries • Accounts for 30% of disease in the US and Europe
Y	• Emerged in the US and also seen recently in South Africa, South America, and Israel • Caused >25% of the disease due to meningococci in the US in the last decade • Causes meningococcal pneumonia in older adults • Responsible for a large proportion of meningococcaemia and meningitis among infants less than 6 months of age
W-135	• Emerged in the last 20 y as a cause of epidemic disease including in South America and Africa • Particularly important in relationship to the Hajj pilgrimage
X	• Recently found to be responsible for meningococcal cases and outbreaks in certain African countries such as Kenya, Niger, and Ghana

### Meningococcal vaccines

The use of meningococcal vaccines is recommended by national and global organizations for the prevention of meningococcal disease.^86,87^ The serogroup C-specific conjugate vaccine has significantly reduced the incidence by 94% of meningococcal disease due to this serogroup in regions where the vaccine is used routinely.^83^ There are currently 2 main types of vaccine used for protection against meningococcal infections; pure polysaccharide vaccines and polysaccharide-protein conjugate vaccines.^88^ Pure polysaccharide vaccines have several important limitations that conjugate vaccines have been designed to help overcome.^89,90^ The most significant problems are that the immunogenic response to pure polysaccharide vaccines is diminished in infants and young children below 2 y of age, they do not prevent nasopharyngeal carriage nor confer herd immunity, and only provide short-term immunity in those older than 2 years, waning after approximately 1–5 y.^89,90^ Meningococcal conjugated vaccines offer a number of advantages including effectiveness in infants, induction of immunological memory and reduced rates of hyporesponsiveness following repeat dosing.^89-91^

The available vaccines include a meningococcal groups A, C, Y and W-135 (MenACWY) polysaccharide diphtheria toxoid conjugate vaccine (Sanofi Pasteur Inc.),^92^ a MenACWY oligosaccharide diphtheria CRM_197_ conjugate vaccine (GSK Vaccines, formerly licensed by Novartis Vaccines and Diagnostics, Inc.)^93^ and the most recently approved quadrivalent conjugate vaccine, MenACWY polysaccharide tetanus toxoid conjugate vaccine (Pfizer, formerly licensed by GSK Vaccines) which received approval in the European Union (EU) in April 2012.^94^ The first broadly effective MenB vaccine for all age groups was approved for use in the EU in January 2013 (GSK Vaccines, formerly licensed by Novartis Vaccines and Diagnostics, Inc.).^95^ Thus, for the first time, there is an opportunity to protect against most of the meningococcal serogroups that cause human disease at the same visit.

### Regional status of meningococcal vaccination

With the exceptions of Bahrain and Saudi Arabia, most countries represented did not include immunization against meningococcal disease in their NIPs at the time of the expert meeting, although meningococcal vaccines were available in the private market in most countries. In Russia, Kazakhstan, Ukraine and several countries in the MENA region, immunization against meningococcal disease is used during outbreaks, but is limited to high-risk groups. Several MENA countries also offer meningococcal vaccination for pilgrims during the Hajj season.

### Barriers to meningococcal vaccine licensure and implementation

The epidemiology of circulating strains was identified as an important consideration for vaccination policy makers, as different meningococcal serogroups are important in different regions. The overall consensus among experts is that vaccine policy makers should consider tetravalent vaccines as these provide the maximum coverage.

Additional barriers identified by the group of experts were the low rates of meningococcal-related morbidity and mortality compared to other diseases such that meningococcal vaccine introduction was considered to have the lowest priority among the vaccines discussed.

A number of key data gaps were also identified in relation to the implementation of routine meningococcal vaccination, which the experts suggested to better understand the need for and benefits of meningococcal vaccination. Several approaches were suggested to help address these gaps including acquiring additional data on the long-term protection offered by conjugated meningococcal vaccines, immunogenicity data in specific age groups (adults over 55 y of age and infants less than 12 months of age), data on interchanging different vaccine types as booster vaccines for infants and more data from studies in which the meningococcal vaccine is co-administered with other vaccines (for adults and children).

## Conclusions

This review summarizes the observations and recommendations from a Pediatric Vaccine-Preventable Diseases Working Group Meeting convened in November 2012. This meeting presented a unique opportunity for experts from developing and developed countries to share their experiences and recommendations for overcoming barriers to vaccine implementation.

In addition to the available literature, the current manuscript provides a summary to local experts and authorities to facilitate decisions for introducing and/or maintaining available pediatric preventions.

Pertussis remains a significant mortality risk for infants ineligible for vaccination and large variation in incidence rates and vaccination strategies exist between regions. Though recommended for use in the EU, there are currently limited data to support the use of cocooning strategies. Several barriers to booster vaccination, including vaccine registration and cost, hence most countries represented suggested an effective completion of the childhood vaccination program to remain the key priority.

Pneumococcal disease remains of key importance across Asia and Africa. High vaccine coverage was considered the most important strategy to reduce the rate of disease, but coverage was low, or non-existent, in most regions. In general, better serotype-specific surveillance data is required to inform vaccine selection and to increase understanding of the importance of serotype replacement and boosters.

Vaccination against measles, mumps and rubella, in conjunction with or independent of varicella was not in use in the public sector in any of the regions represented at the time of the meeting. This was felt to have been influenced by the wording of current European and American guidance. The inclusion of more relevant efficacy and cost-effectiveness data, a simplification of the wording and greater acknowledgment of region-specific data would make recommendation more meaningful and applicable. Overall, better communication of the benefit/risk ratio by providing parents with leaflets and product insert to read this information was considered to be an important step toward implementation of large-scale vaccination programmes.

With regard to rotavirus, education regarding the health burden and impact of infection, and the sharing of current data sets, were considered important to emphasize the benefits of vaccination. Experts agreed that the benefits of rotavirus vaccination outweighed the risk of intussusception. Benefit/risk analyses, improved surveillance and measures of cost effectiveness will play a key role in future recommendations.

The need for risk and benefit of vaccination against meningococcal disease was felt to be less well understood by policy makers and due to limited uptake, few data were available for interrogation.

In conclusion, the burden of pediatric vaccine-preventable disease remains high in developing nations. The currently available pediatric vaccines are highly efficacious with a favorable benefit/risk profile, and have the potential to greatly reduce this disease burden. Various methods of overcoming barriers to vaccine implementation in the future were proposed. These included: improving diagnosis and surveillance methods, improving education and communication about pediatric diseases, and developing more relevant pediatric vaccination guidelines.

## Abbreviations

AAP: American Academy of Pediatrics
ACIP: Advisory Committee on Immunization Practices
aP: acellular pertussis
B-CAP: bacterial community-acquired pneumonia
CDC: Centres for Disease Control and Prevention
CIS: Commonwealth of Independent States
COPE: Consensus on Pertussis Booster Vaccination in Europe
DTP: diphtheria, tetanus, pertussis
EU: European Union
GAVI: Global Alliance for Vaccines and Immunization
HCP: health care provider
IPD: invasive pneumococcal disease
IS: intussusception
MENA: Middle East and North Africa
MMRV: measles, mumps, rubella, varicella
NIP: national immunization programme
PCR: polymerase chain reaction
PCV: pneumococcal conjugate vaccine
RVGE: rotavirus gastroenteritis
STIKO: German Standing Committee on Vaccination
Tdap: reduced antigen concentration tetanus, diphtheria, acellular pertussis
UMV: universal mass vaccination
WHO: World Health Organization
wP: whole cell pertussis
